# Associations between hair trace mineral concentrations and the occurrence of treponeme-associated hoof disease in elk (*Cervus canadensis*)

**DOI:** 10.1186/s12917-022-03547-3

**Published:** 2022-12-23

**Authors:** Steven N. Winter, Maria del Pilar Fernandez, Kyle R. Taylor, Margaret A. Wild

**Affiliations:** 1 Department of Veterinary Microbiology and Pathology, Washington State University, Pullman, WA 99164 USA; 2 Paul G. Allen School for Global Health, Washington State University, Pullman, WA 99164 USA; 3 Washington Animal Disease Diagnostic Laboratory, Washington State University, Pullman, WA 99164 USA

**Keywords:** Elk, *Cervus canadensis*, hair, trace minerals, treponeme-associated hoof disease

## Abstract

**Background:**

Trace minerals are important for animal health. Mineral deficiency or excess can negatively affect immune function, wound healing, and hoof health in domestic livestock, but normal concentrations and health impairment associated with mineral imbalances in wild animals are poorly understood. Treponeme-associated hoof disease (TAHD) is an emerging disease of free-ranging elk (*Cervus canadensis*) in the U.S. Pacific Northwest. Selenium and copper levels identified in a small number of elk from areas where TAHD is established (i.e., southwestern Washington) suggested a mineral deficiency may have increased susceptibility to TAHD. Our objectives were to determine trace mineral concentrations using hair from elk originating in TAHD affected areas of Washington, California, Idaho, and Oregon and assess their associations with the occurrence of the disease.

**Results:**

We identified limited associations between TAHD occurrence and severity with hair mineral concentrations in 72 free-ranging elk, using Firth’s logistic regression and multinomial regression models. We found consistent support for a priori hypotheses that selenium concentration, an important mineral for hoof health, is inversely associated with the occurrence of TAHD. Less consistent support was observed for effects of other minerals previously associated with hoof health (e.g., copper or zinc) or increased disease risk from potential toxicants.

**Conclusion:**

Trace mineral analysis of hair is a non-invasive sampling technique that offers feasibility in storage and collection from live animals and carcasses. For some minerals, levels in hair correlate with visceral organs that are challenging to obtain. Our study using hair collected opportunistically from elk feet submitted for diagnostic investigations provides a modest reference of hair mineral levels in elk from the U.S. Pacific Northwest that may be useful in future determination of reference ranges. Although our results revealed high variability in mineral concentrations between elk, consistent relationship of possibly low selenium levels and TAHD suggest that further investigations are warranted.

**Supplementary Information:**

The online version contains supplementary material available at 10.1186/s12917-022-03547-3.

## Background

Minerals are important in the maintenance of animal health. Adequate concentrations of minerals such as zinc (Zn), selenium (Se), and copper (Cu) are essential for optimal immune function [1, 2]. In domestic ruminants, these minerals, as well as others including manganese (Mn [3]) and magnesium (Mg [4]), also contribute to the quality and health of hooves. Cattle with deficiencies in these minerals are more prone to lameness and foot diseases that may be mitigated with mineral supplementation [5]. The underlying disease mechanisms are likely multifactorial and may include effects of minerals on keratinization and exfoliation of hooves and skin [6], immune response and oxidative stress [7], and gut microbiome [8]. Conversely, heavy metals such as arsenic (As) or chromium (Cr [9]) can act as toxicants. Exposure to pollutants, for example from industrial output or pesticides, may result in unhealthy accumulation in animals [10, 11].

Treponeme-associated hoof disease (TAHD) is an emerging disease of free-ranging elk (*Cervus canadensis*) in the U.S. Pacific Northwest [12]. Elk with TAHD exhibit interdigital and sole ulcers, overgrown and deformed hooves, and sloughed hoof capsules [13]. Although characteristic spirochetes present in lesions have been identified as *Treponema* spp. similar to those observed in cattle with bovine digital dermatitis [13, 14], the definitive cause is not yet established. Because of the relationship of mineral levels to hoof diseases in cattle, and potentially low hepatic Se and Cu levels observed in a limited number of elk examined from a TAHD-established area of southwestern Washington, U.S., investigators have speculated that deficiency may contribute to susceptibility to TAHD [13, 15].

Investigating the effects of mineral concentration on disease status of free-ranging wildlife is challenging. A paucity of information exists on the normal ranges of mineral concentrations for wild ruminants such as elk. As a result, normal levels for cattle or samples of free-ranging animals from select areas are often used as reference values [16, 17]. These values likely do not represent normal ranges in elk. Further, concentrations likely vary with age, along with the broad geographic range that elk occupy and the associated differences in soil, diet, climate effects on forage, and presence of contaminants [18, 19]. An additional challenge is access to organs, such as liver, for mineral analysis in wildlife because of complexities in collection, storage, and transport of field samples for submission. Hair analysis has been used as an alternative by some researchers to determine the mineral status of a range of wildlife species (reviewed by [19]). Recent research on red deer (*C. elaphus*) [10] and woodland caribou (*Rangifer tarandus caribou*) [20] found multiple trace elements derived from hair were strongly correlated with concentrations in visceral organs. Hair is easily collected from live animals or at postmortem and does not require special handling for stability of the sample [20, 21]; it is also opportunistically available in samples submitted for wildlife disease surveillance (e.g., feet for TAHD, or heads for chronic wasting disease or bovine tuberculosis surveillance).

Our objectives were to determine and provide baseline information on hair mineral concentrations found in free-ranging elk from the U.S. Pacific Northwest, and to assess associations between mineral concentrations and the occurrence of TAHD. Findings from descriptive explorations promote understanding of susceptibility of elk to TAHD and encourage hypothesis generation [22] for future studies.

## Results

**Fig. 1:**
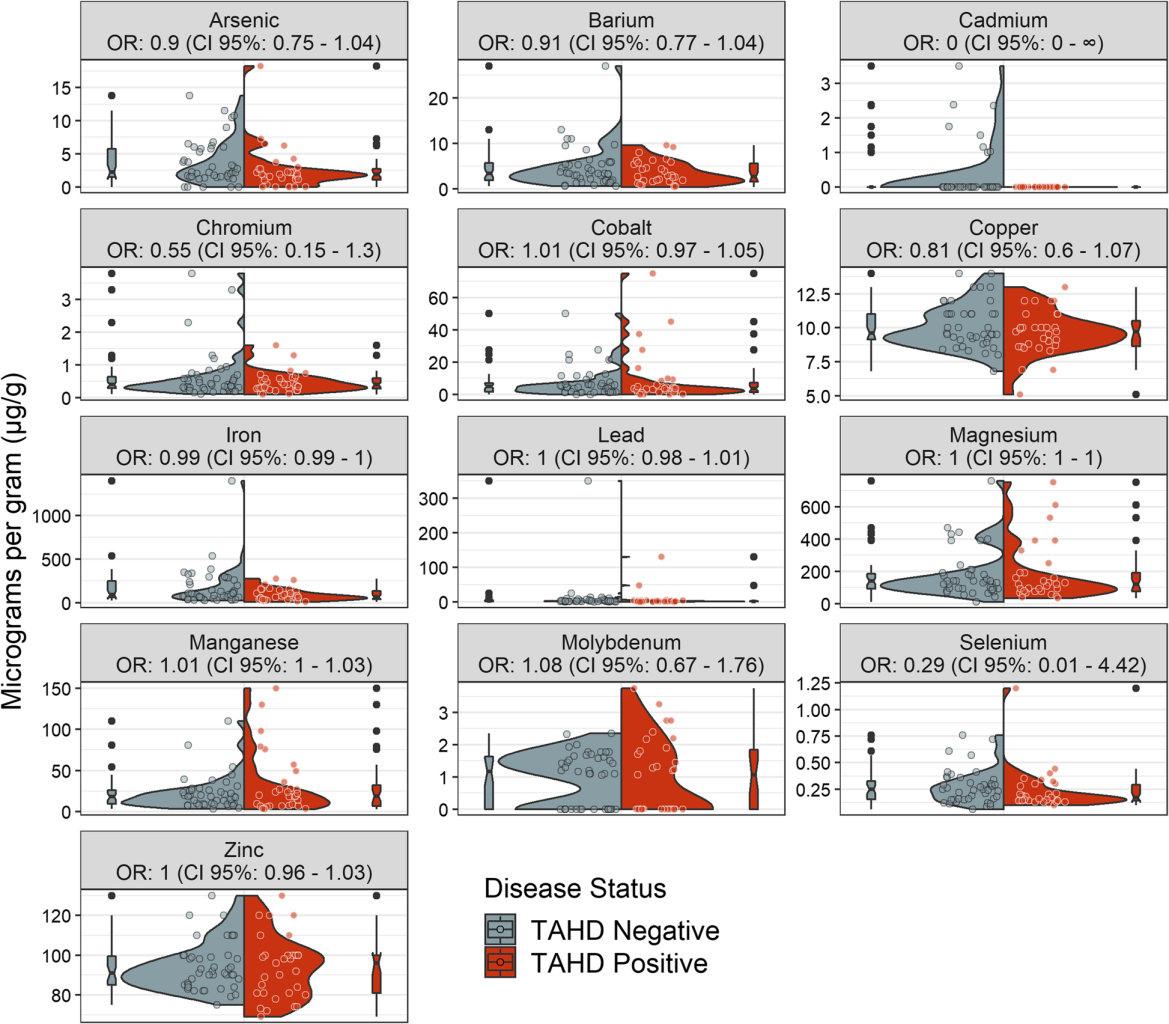
Distributions of mineral concentrations and univariate odds ratios with respect to treponeme-associated hoof disease (TAHD) diagnosis. Split violin plots show distributions of mineral concentrations (μg/g) scaled specific to the 13 elements (panel labels) assessed in hair analyses in elk while overlaid colored points represent raw data for each disease status: TAHD negative (gray colors) and positive (red colors). Univariate logistic regressions generated simple odds ratios (OR) and 95% confidence intervals (CI) for minerals. Boxplots at the peaks of violin plots show the median and 25th and 75th interquartile ranges while black dots represent outliers. Of trace minerals relevant to hoof health, we noted two outlier values in selenium and cobalt. Arsenic, cadmium, cobalt, lead, and molybdenum values were below reporting limits; values shown are rescaled (see Methods: Data pre-processing section). Raw values for all minerals are in a data repository [23]

We report the concentration of 13 analyzed minerals from hair and relevant surveillance metadata in a publicly available data repository [23]. We observed qualitatively similar distributions in hair mineral concentrations from TAHD positive and negative elk for most minerals (Fig. 1).

Mann-Whitney tests identified significant differences between values from TAHD positive and negative cases for only two elements: cadmium (Cd) [Mann-Whitney *U* = 542, *p *= 0.01], which had perfect separation with identical values of 0 μg/g for all TAHD-positive cases, and lead (Pb) [Mann-Whitney *U* = 447, *p *= 0.02]. Two elk had uncharacteristically high values for Se and cobalt (Co) (Fig. 1) garnering consideration as outliers. Candidate datasets omitted either the Se or Co outlier (*n *= 73) or both elk (*n *= 72). While having similar classification performance (as indicated by the area under the model’s receiver operating characteristic curve, or AUC), our dataset selection approach favoring more balanced sensitivity and specificity supported the removal of both Se and Co outliers (Table S1).

Preliminary data explorations revealed varying strengths of correlations between minerals assessed in hair analysis (Fig. S2), and as such, we reduced a priori analyses to only those elements without strong correlations (*ρ* < |0.7|) but still relevant to hoof health (i.e., Se, Co, Cu, Mo, and Zn [5]) or toxicant-related hypotheses (i.e., As, Cr, Co, and Pb [24]) (Table 1).

**Table 1:** Ten hypotheses of mineral influences on treponeme-associated hoof disease (TAHD) in elk. We evaluated multiple models to address independent a priori hypotheses and post hoc models on hoof health minerals and toxicants’ influence on TAHD diagnosis and severity of disease (i.e., “Status” and “Grade_Severity”, respectively) using the dataset without outliers. Model formulas are in italics beside a shorthand for their motivating hypotheses

Model	Formula
**A priori hypotheses**	
A. Hoof health minerals	Status ~ Co + Cu + Mo + Se + Zn
B. Hoof health minerals with sex adjustment	Status ~ Co + Cu + Mo + Se + Sex + Zn
C. Hoof health minerals with “Longitude” ^a^ adjustment	Status ~ Co + Cu + Mo + Long. + Se + Zn
D. Toxicant hypothesis	Status ~ As + Co + Cr + Pb
E. Toxicants with “Longitude” adjustment	Status ~ As + Co + Cr + Long.+ Pb
F. Severity of disease and hoof health minerals	Grade_Severity ~ Co + Cu + Mo + Se + Zn
G. Severity of disease affected by toxicants and “Longitude”	Grade_Severity ~ As + Co + Cr + Long.+ Pb
**Post hoc models**	
H. Interaction^b^ of “Longitude” and Se	Status ~ Co + Cu + Mo + Se * Long. + Zn
I. Se with toxicants and “Longitude” adjustment	Status ~ As + Co + Cr + Long.+ Pb + Se
J. Cr (as possible toxicant) exacerbating Se deficiency	Status ~ Co + Cu + Mo + Se * Cr + Zn
K. Co removed and substituted with Mn from Model C	Status ~ Cu + Long. + Mo + Mn + Se + Zn

^a^Longitude category = “west” or “east” assigned from available location information (see Methods)

^b^Interaction term was applied with releveling longitude under two sub-models: H1 and H2

**Fig. 2:**
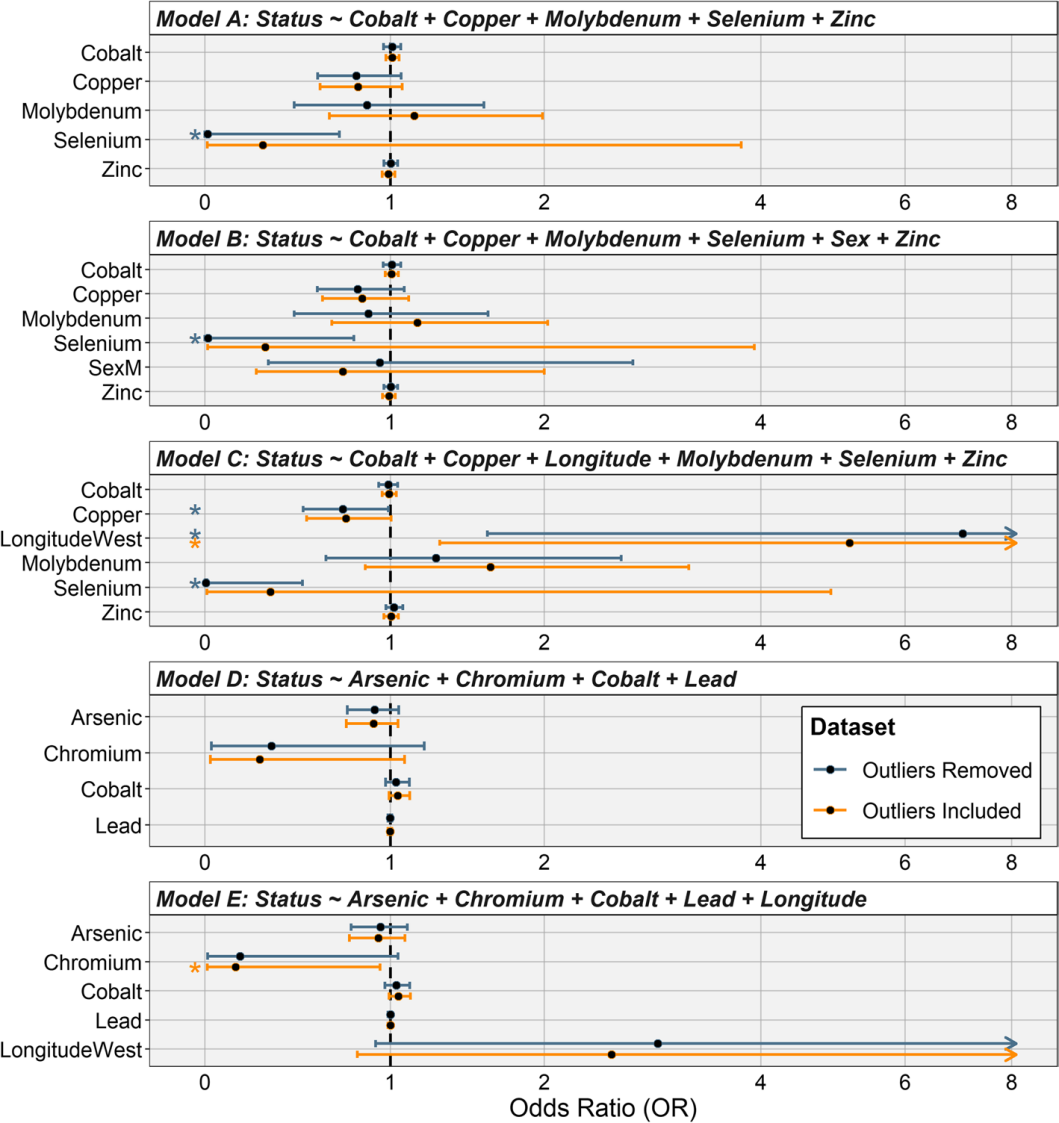
Explanatory variable results from addressing five a priori hypotheses. Five panels show different models with visual summaries of explanatory variables’ estimates of odds ratios (black circles), 95% confidence intervals (bars), and presence of a statistically significant relationship (denoted by a star, ⋆) color-coded to represent different datasets: outliers removed (blue) or outliers included/full dataset (orange)

Among the models exploring the minerals important for hoof health and TAHD disease status (Models A-C), we observed a significant inverse relationship between Se concentration and TAHD (Se odds ratios [ORs] ≈ 0.01; confidence intervals [CIs] 95%: [<0.01 – 0.79]; *p* ≈ 0.03) (Fig. 2). We noted similarly inverse trends for Cu (ORs ≈ 0.78), which were significant (*p* = 0.04) when “Longitude” was included (Model C)*.* By contrast, Mo concentrations were positively correlated to TAHD when “Longitude” was included in the model; this relationship, however, was not statistically significant. In toxicant-related models (Models D and E), Cr and As concentrations showed an inverse trend with the odds for TAHD, but only the association with Cr was statistically significant after adjusting for “Longitude” (Fig. 2). Other variables (i.e., male sex, Co, Zn, Pb) had ORs ≈ 1 and CIs marginally moved about their point estimates when other covariates were included, suggesting little to no evidence of relationships (*p* > 0.05).

A priori model comparisons indicated that models exploring the association between heavy metal toxicants (Table 1, Models D-E) had a better fit to the data than models exploring the association with hoof health (Models A-C), based on Akaike’s information criterion with small sample size adjustment (AICc) values (Table 2); however, their classification performance was similar, with sensitivity and specificity varying widely between models. Model D had modest sensitivity (0.76) but poor specificity (0.56); Model E, which included “Longitude”, had similarly poor specificity (0.53), but was nearly twice as sensitive (0.90). In agreement with the most frequent distribution of cases in the west versus the east in this region, the best fitting models included “Longitude” as an explanatory variable and warranted its inclusion (Table 2, Fig. 2). We observed many explanatory variables’ CIs for ORs were sensitive to the presence of outliers, but OR point estimates were relatively consistent. All explanatory variables’ variance inflation factor were ≤ 2, so no multicollinearity issues were observed. Finally, multinomial regressions (Models F and G) revealed generally consistent relationships observed in earlier models (Fig. S3), but we did not identify any statistically significant associations between mineral concentrations and grade severity category.

**Table 2:** Model comparisons for logistic regression models. Alike models were compared by goodness of fit and performance. Note only models using similar response variables were compared and evaluation of two multinomial models can be found in supplementary materials

Model	AICc^**a**^	AUC^**b**^	Sensitivity	Specificity^**c**^
**A priori hypotheses**				
A. Hoof health minerals	86.83	0.67	0.93	0.42
B. Hoof health minerals with sex adjustment	88.06	0.67	0.93	0.42
C. Hoof health minerals with “Longitude”^c^ adjustment	88.46	0.74	0.76	0.65
D. Toxicant hypothesis	80.51	0.65	0.76	0.56
E. Toxicants with “Longitude” adjustment	81.73	0.70	0.90	0.53
**Post hoc models**				
H. Interaction of “Longitude” and Se	93.55	0.77	0.72	0.72
I. Se with toxicants and “Longitude” adjustment	85.30	0.74	0.72	0.74
J. Cr (as possible toxicant) exacerbating Se deficiency	93.39	0.70	0.76	0.58
K. Co removed and substituted with Mn from Model C	86.49	0.74	0.69	0.70

^a^*AICc *Akaike information criterion for small sample sizes

^b^*AUC *Area under the receiver operating characteristic curve

^c^Longitude category = “west” or “east” assigned from available location information (see Methods)

We further evaluated four models post hoc to challenge observed effects of Se, As, and Cr. Model H evaluated whether Se exhibited a dose-dependent effect with “Longitude” under each of the reference categories of the “Longitude” variable (west -Model H1- and east -Model H2-; Fig. S4). We found a dose-dependent effect only when outliers were included, mostly driven by a TAHD positive elk with a very high level of Se in the east. Model I *–* an extension of the toxicant-related Model E with an additional Se term – had relatively similar OR point estimates with respect to toxicants, but CIs expanded for both Se and “Longitude” beyond 1 (*p* > 0.05) (Fig. S4). We also added an interactive effect between Cr and Se for Model J to explore whether Cr may exacerbate low Se values; however, this interactive term between the two minerals did not yield a clear nor statistically significant effect (Fig. S4). Finally, as Mn can also be important for hoof health [5] but was strongly correlated with Co (*ρ* = 0.8; Fig. S2) included in initial models, we substituted Co for Mn in Model K, but noted little to no influence on odds for TAHD (OR ≈ 1), consistent with the univariate model (Fig. 1).

## Discussion

We report hair mineral concentrations from a regional sample of elk, improving available data for developing reference ranges for mineral measurements in free-ranging elk [25]. We found evidence that relatively higher Se values were less likely in elk with TAHD, consistent with some studies noting reduced severity in other ruminant hoof diseases when Se is supplemented [26, 27]. Contrary to our hypotheses, however, we observed no consistently significant associations with additional minerals important to hoof health, as well as an inverse relationship between potential toxicants and TAHD. Some associations may have been obscured by the relatively small size of our dataset, unexplainable variation from age which was omitted due to missing metadata, and other important considerations discussed below. Still, if TAHD was clearly exacerbated by relative excess or deficiency of specific minerals, we would expect to see strong relationships among these initial models despite the dataset’s size. These results emphasize a need for future sampling designs to continue investigating associations between mineral concentrations and TAHD across the affected region.

In agreement with expectations and previous reports of free-ranging elk in a TAHD-established area, we found that TAHD was less likely to occur in elk with higher Se values, as indicated by ORs < 1. Selenium’s relationships (via OR point estimates) were consistent across multiple models that adjusted for potentially confounding variables. As hair has been regarded as a good indicator of body Se [28], these associations could be evidence of Se having a protective effect on disease susceptibility. Although more research is needed, this interpretation would be consistent with some observations of reduced severity in other ruminant hoof diseases when Se was supplemented in livestock [26, 27, 29, 30].

Contrary to our expectations, however, we noted mixed to limited evidence for relationships between TAHD and several other minerals important for hoof health. We found evidence for increased Cu levels in hair having a significant inverse association with TAHD only when adjusting for geographic confounders. This relationship agrees with low Cu levels found in elk from TAHD-affected areas [13, 15, 17] and the mineral’s importance in cattle with digital dermatitis [5]. Copper's change in significance indicated there were probably different Cu levels in elk sampled in the east and the west, and its effects were not clarified until that variation was explained by the “Longitude” proxy variable. Although reduced Zn is associated with lameness and digital dermatitis in cattle [5, 31], we detected neither strong nor significant negative correlations between TAHD and Zn in hair, and levels were within normal ranges for cattle [32]. These results were consistent with past studies that reported normal hepatic Zn levels in elk from TAHD-established areas [13, 15].

We hypothesized that As and Cr concentrations may be associated with potential herbicide or toxicant exposure [10, 11] and would positively correlate with disease, consistent with a toxicant-mediated infection from theoretical bases [33]. However, we found an inverse association between TAHD and these minerals and a lack of a significant dose-dependent effect between Cr and Se (i.e., increasing Cr did not appear to exacerbate lower Se concentrations). Toxicants’ apparent protective effects remained despite adjusting for “Longitude” in Model E. Interestingly in post hoc Model I, adding a Se term to the model aimed to challenge a toxicant-related hypothesis expanded the CIs for both “Longitude” and Se – a sign suggestive of redundant information between Cr, Se, and “Longitude” for disease status.

We expected lower hoof health mineral concentrations and/or higher toxicant concentrations would be positively correlated with major lesions. We hypothesized that lower concentrations of hoof health minerals might facilitate lesion progression or mineral availability may be reduced by sequestration following sustained inflammation [34] while toxicants may exacerbate existing infections [33]. Contrary to expectations, we did not detect statistically clear associations in mineral concentrations between lesion severity categories. We suspect failure to identify a statistically significant effect could be related to sample size limitations, and thus, additional samples may be needed to address these hypotheses.

Although some observed TAHD-mineral relationships correspond with biological bases, others may be artifacts from sampling locations, our modest sample size, or limited value in hair’s use to measure mineral concentrations. For instance, with respect to Se, Cu, and some toxicants (As and Cr), observed inverse associations could also be possible if low mineral concentrations were present coincidentally in otherwise high-risk locations where transmission is high, or if high mineral levels were present in populations unexposed to TAHD due to unrelated factors (e.g., barriers to spread). Besides sampling considerations, the size of the dataset may also be responsible for many models having considerable variation in explanatory variables’ CIs and OR point estimates before and after removing outlier values. We suspect the size of the dataset is responsible for the pronounced variation in models assessing grade severity (i.e., Model F and G). Finally, it cannot be excluded that hair may have limited measurement value for some minerals. For example, pairwise correlations did not detect a strong relationship between Zn and Cu (*ρ *= – 0.1) in our dataset despite physiologic competition for gastrointestinal absorption. This was surprising because previous reports of *C. elaphus* hair competently revealed Zn-Cu relationships that correlated with organ concentrations [10, 35]. It is unclear why this relationship was not detected, but this could be possible if hair was a suboptimal medium for evaluating Zn and Cu levels, as found in Canadian caribou [20], or if lower Zn levels were related to the body location from where hair was sampled, as seen in cattle [36]. Similarly, hair simply may not represent toxicant exposures in our study area as well as reported for European red deer [11, 35]. Given unclear availabilities of Se and other minerals in forage, and the complex influences of forestry practices on elk nutrition and forage selection [37], it is difficult to disentangle mineral’s effects on TAHD with available information.

Resolving ambiguities in geographic sampling locations and the measurement value of hair will be challenging in natural settings as broad regions (i.e., coastal Pacific Northwest) are often declared deficient with respect to nutrient availabilities (e.g., Cu or Se [17, 28]). Further, a disconnect exists between mineral concentrations found in soil distribution maps and what is accessible to herbivores in forage, much less elk preferences (or lack thereof) for these plants, including Se containing and accumulating plants [38]. More balanced sampling from mineral rich and sparse areas as well as controlled challenge studies will be needed to understand the true effects of minerals on TAHD pathogenesis. Such controlled studies could also assess mineral concentrations prior to and following the onset of TAHD to avoid uncertainties that arise when comparing single point in time values between apparently healthy and diseased elk.

Results from studies in livestock are often extrapolated to guide disease management in wildlife. Supplementation with some mineral formulations may decrease incidence and severity of digital dermatitis in cattle [26, 39] suggesting that similar treatment may be warranted to manage TAHD in free-ranging elk. However, at least until further studies are conducted, we urge caution in implementing such an action for several reasons. First, current information is not available to confirm that occurrence or severity of TAHD in elk would be reduced with mineral supplementation, particularly standard formulations. For example, although we could find no published reports of mineral concentrations in sheep with contagious ovine digital dermatitis, a treponeme-associated hoof disease similar to TAHD of elk, sheep with footrot exhibited lower Se concentrations similar to elk in our study; however, supplementation of sheep with Se did not prevent or improve recovery from footrot [27]. Second, unlike diets provided to livestock or captive wildlife in which mineral availability can be readily manipulated, mineral supplementation is logistically challenging if not unfeasible for free-ranging wildlife. This is particularly true over widespread natural areas. Finally, and perhaps most importantly, a management action, such as supplementation via mineral blocks, could amplify disease risks. TAHD is a transmissible infectious disease [40] so congregating elk may inadvertently promote TAHD transmission through increased exposure to pathogens [41].

Although our work presents interesting findings, careful interpretation is warranted. Namely, these hair samples are cross-sectional – simple snapshots of diet, body condition, and physiological state– that offer only some insight to organ mineral concentrations and biological availability [10, 20, 21]. As snapshots, it is unclear whether observed mineral changes contributed to TAHD or vice versa, thus presenting a “chicken or the egg” conundrum, should a direct cause and effect exist. For example, it would be unclear whether diminished Se increases susceptibility to or progression of this infectious disease, or whether Se decreases as a result of TAHD infection and immune-related costs [34]. Such obscurities are not easily resolved given individual-level variation and limited knowledge of mineral availabilities at the host-pathogen interface as well as possible mismatches in concentrations between hosts and environments [34]. Explorations of other environmental drivers will be needed to understand factors that contribute to TAHD susceptibility and risk explicitly, which will provide useful information for conducting wildlife disease surveillance and management.

## Conclusion

Mineral concentrations in wildlife are important contributors to health but are complex to interpret due to species and regional differences and limited clarity for what can be considered “normal.” We present a regional analysis of preliminary linkages between mineral concentrations found in free-ranging elk and an emerging disease of conservation concern. In addition to supporting some existing hypotheses, our work revealed some unexpected results that require careful consideration prior to advisement of any management interventions (e.g., mineral supplementation). Balanced surveillance programs, controlled deficiency- or toxicant-related challenge studies, and local-level environmental sampling will be needed to further disentangle these effects. Ultimately, if mineral imbalances are confirmed, mitigation techniques that do not concurrently increase risk of pathogen transmission will be needed to successfully manage disease in free-ranging elk.

## Methods

### Sample collection

Hooves were collected postmortem from Roosevelt (*C. c. roosevelti*) and Rocky Mountain (*C. c. nelsoni*) elk as part of surveillance efforts for TAHD using techniques similar to those previously described [12]. Hooves from free-ranging elk harvested or collected by state, tribal, and federal wildlife managers in 2017-2021 were submitted to the Washington Animal Disease Diagnostic Laboratory (Pullman, Washington). Normal and deformed hooves were obtained from states where TAHD is known to affect elk populations, including Washington (*n *= 41), Oregon (*n *= 6), Idaho (*n *= 13), and California (*n *= 14), U.S. (Fig. S1). A board-certified veterinary pathologist assigned a gross lesion score (grading) and conducted histologic evaluation of submitted hooves. Briefly, gross TAHD lesions in early stages progress from ulcerations and erosion of the interdigital space (grades I and II) to ulceration of the sole/heel-sole junction and sloughing of hoof capsules (grades III and IV, respectively); for details see [13, 40]. A diagnosis of TAHD was made based on the presence of suppurative inflammation and invasive spirochetes on histology [13]. We omitted from analyses submissions with incongruencies between gross and histologic lesions to retain only confirmed positive and negative cases (*n *= 74).

### Hair analysis

Because the body location from where hair is sampled can influence results [42], we clipped approximately 2 g hair consistently from the pastern area of one foot from each elk submitted for TAHD surveillance. Hair was kept frozen at -29 to -62 C until analysis. Hair samples were washed with acetone to remove surface contaminants [43], and analyzed for a selected panel of 13 trace minerals (i.e., As, Ba, Cd, Cr, Co, Cu, Pb, Fe, Mg, Mn, Mo, Se, Zn) by the University of Idaho Analytical Sciences Laboratory (Moscow, Idaho) using inductively coupled plasma mass spectrometry (ICP-MS). Hair mineral concentrations were reported in micrograms per gram (μg/g) of dry hair.

### Data pre-processing and descriptive analyses

Minimum reporting limits were specific to each mineral. Given that some values were below the reporting limits, we rescaled values by assigning those less than the limit as zero and dividing values at or above the limit by the reporting limit to quantify relative increases in mineral concentrations. That is, when a mineral had a detection limit of 0.040 μg/g, for example, measurements recorded as < 0.040 were re-assigned as zero, and all values ≥ 0.040 were divided by 0.040. Because mineral concentrations might be highly correlated [35], we explored correlations using principal components analysis and calculating Spearman’s rank correlation coefficients to assess the association between pairwise mineral concentrations. We calculated ORs and CIs from logistic regression models examining individual mineral concentrations’ relationships with positive diagnosis of TAHD. Finally, we stratified data by TAHD diagnosis, plotted distributions of mineral concentrations, and assessed for significant differences in their values using Mann-Whitney tests.

We merged results from hair analysis with additional surveillance metadata at the individual elk level. Considering limited environmental availability of some minerals in parts of the region (e.g., Se or Cu [16, 17, 44]) and focal areas of increased cases/surveillance [12], defining the regions from where elk samples originated was necessary. However, several variables (e.g., precise latitude/longitude, game management unit of harvest) had missing entries that could not be imputed using other information available and could not be included in analyses. Rather than omitting or misattributing spatial properties of data, we used available information to conservatively reclassify cases into a broad scale longitudinal category variable because of the importance of longitude in mineral concentrations [44]. This variable also corresponded with elk ecotype from other investigations [45] considering known patterns of Roosevelt elk generally being found along the Pacific Northwest coast, while Rocky Mountain elk can be found east of the Pacific crest and into Idaho [46, 47]. For clarity, we named this variable “Longitude” to represent its use as a crude east-west dichotomy rather than  directly attributing it to intrinsic biologic difference in ecotype, although this cannot be excluded. Finally, we grouped cases based on the grade of hoof lesions observed using Han et al.’s classification scheme [13]. We used the highest grade observed in a case to assign the individual into one of three categories to define disease severity (termed “Grade_Severity”). Specifically, highest grade present among hooves graded as zero were considered “Zero”, grades I-II as “Minor”, and grades III-IV as “Major.”

### Modeling strategy

We used multiple regression models to explore associations between TAHD and mineral concentrations. When binary disease diagnosis status (0 = TAHD negative, 1 = TAHD positive) was the dependent variable, we used Firth’s bias reduction [48] with the logistf package [49] in R (v 4.0.5.). When the dependent variable had more than two categories (i.e., categories of hoof lesion severity), we built multinomial regression models using the brglm2 package [50]. Although mechanistically distinct, both methods penalize bias in maximum likelihood estimators, producing finite parameter estimates and reducing separation common in datasets with small sample sizes or unbalanced data [51, 52].

We assessed support for different a priori hypotheses (Models A-G; Table 1) assessing associations between TAHD (i.e., binary diagnostic status or categorical severity of graded lesions) and minerals important for hoof health, potential environmental toxicants [24], as well as geographic or sex-specific confounding variables. Briefly, we hypothesized that minerals important for hoof health (e.g., Zn, Se, Cu, Mo, Co) would inversely correlate with TAHD status (Model A). Given sex-specific nutritional demands (e.g., parturition, antler development [53]), we suspected these hoof health minerals may be additionally confounded by sex (Model B). Also, surveillance effort has been historically focused in western Washington [12]; thus, adjusting for longitude may influence effects of hoof health minerals (Model C). Next, as hair may be a useful metric for identifying concentrations for heavy metal toxicants [10], we expected that elements often associated with herbicide chemicals would positively correlate with TAHD status [33] (Model D). However, as private and publicly owned forested elk habitat in western Washington, Oregon, and parts of northern Idaho are subjected to varying forestry management practices (including use of silvicultural herbicides) [37], adjustment for longitude was explored (Model E). Finally, with respect to lesion severity, we hypothesized that lower mineral concentrations (Model F) and/or higher toxicant concentrations (Model G) would be associated with elk having major lesions.

From the limited sample size, we suspected outliers in data potentially originating from measurement errors may distort relationships. Hence, we plotted mineral distributions with respect to TAHD diagnosis to identify any outliers. To understand how outliers influenced analyses, we developed subset datasets from the full dataset removing samples that had uncharacteristically high mineral values (Co and Se). We compared these datasets using a simple model (Model A) and evaluated how each dataset influenced Model A’s performance (i.e., AUC). We used both the complete dataset and the best performing candidate dataset (termed as the “outliers removed” dataset) with the highest AUC considered with respect to the most balanced sensitivity-specificity ratio in our modeling strategy (Table S1**)**. We used AICc [54] in the MuMIn R package [55] to identify the best fitting model(s) given the data available. We assessed models for multicollinearity issues using variance inflation factor and interpreted explanatory variables’ relationships using ORs and 95% CIs of point estimates. Finally, we created four additional post hoc models after testing our initial hypotheses to assess the consistency and validity of inferences observed in trace minerals (i.e., Se, As, and Cr). Briefly, post hoc models evaluated: hoof health minerals and a dose-dependent response of Se with “Longitude” (Table 1, Models H1 and H2), effects of including a Se term to Model E (Model I), assessing whether Cr may exacerbate a Se deficiency (Model J), and a variant of Model C replacing Co for Mn – another mineral vital for hoof health but correlated with Co.

## Supplementary Information

**Additional file 1: Table S1.** Relative model performance given candidate datasets. We evaluated changes in relative model performance using the same model applied to different datasets with and without notable outliers removed. Note the simple model had higher area under the receiver operating characteristic curve (AUC) when outliers were removed, but sensitivity and specificity were more balanced when both outliers were removed. **Figure S1**. County-level distribution of elk hair samples for mineral analyses. The number of samples analyzed for mineral concentrations from counties are represented by a color gradient, with dark purple colors for one to a few samples and brighter yellow colors for many samples (> 10). We included the Cascades Mountain range (gray polygon) to serve as a landmark commonly used to differentiate where Roosevelt elk (west of cascades) and Rocky Mountain elk (east of cascades) are found. This differentiation was used as the proxy for a longitudinal gradient in models. Most samples were collected in Washington (*n *= 41) followed by California (*n *= 14), Idaho (*n *= 13), and Oregon (*n *= 6). Samples missing specific county data (*n *= 6) were omitted from visualization, but generally, five samples from Washington originated west of the Cascades (west) and one sample came from northern Idaho (east). **Figure S2.** Correlation plots and principal components analysis (PCA) biplot of available trace minerals. Left: Colored square panels show pairwise Spearman’s rank correlation coefficients (ρ) for all mineral pairs. Co-Mg, Co-Fe, Co-Mn, and Mn-Mg were the combinations with strong (≥ |0.7|) correlation values. Right: Results from PCA identifies overlapping ellipses for each U.S. state (colored ellipses) while bidirectionality in loadings suggests closely aligned vectors have similar variance structures and may be correlated (e.g., chromium and cobalt). **Figure S3.** Multinomial models for lesion severity in 20 TAHD positive elk. Two panels show results from multinomial models assessing severity of lesions detected: Grades I and II = Minor (yellow) and Grades III and IV = Major (pink). Odds ratios (OR; black dots) and 95% confidence intervals are graphically represented in each panel for models addressing different hypotheses (text in bold). Potentially due to the small sample size (*n *= 20), we found no statistically significant evidence for odds of grade severity being influenced by minerals included under the models. **Figure S4.** Results from post hoc models. Five panels show four different models and visual summaries of explanatory variables’ estimates of odds ratios (black circles), 95% confidence intervals (bars), and presence of a statistically significant relationship (denoted by a star, ***⋆***) color-coded to represent different datasets: outliers removed (blue) or outliers included/full dataset (orange). Please note Models H1 and H2 are identical models with different references used in the categorical Longitude variable. That is, Model H1 assesses a selenium interaction with respect to the west, while Model H2 assesses the east.

## Data Availability

All mineral data and associated surveillance metadata used in this study are available on an openly accessible data repository (22; 10.5281/zenodo.6964424). Any additional study-related data will be made available from the corresponding author upon reasonable request.

## Abbreviations

TAHD: Treponeme-associated hoof disease
CI: Confidence interval
OR: Odds ratio
AIC_C_: Akaike information criterion with small sample size correction
AUC: Area under the receiver operating characteristic curve
As: Arsenic
Ba: Barium
Cd: Cadmium
Co: Cobalt
Cr: Chromium
Cu: Copper
Fe: Iron
Mg: Magnesium
Mn: Manganese
Mo: Molybdenum
Se: Selenium
Zn: Zinc
