# Supplementary material for: Associations between hair trace mineral concentrations and the occurrence of treponeme-associated hoof disease in elk (Cervus canadensis)
Source: BMC Vet Res. 2022 Dec 23;18:446. doi: 10.1186/s12917-022-03547-3 (PMC9783704; doi:10.1186/s12917-022-03547-3)
Supplement: Supplementary file 1 — Additional file 1: Table S1. Relative model performance given candidate datasets. We evaluated changes in relative model performance using the same model applied to different datasets with and without notable outliers removed. Note the simple model had higher area under the receiver operating characteristic curve (AUC) when outliers were removed, but sensitivity and specificity were more balanced when both outliers were removed. Figure S1. County-level distribution of elk hair samples for mineral analyses. The number of samples analyzed for mineral concentrations from counties are represented by a color gradient, with dark purple colors for one to a few samples and brighter yellow colors for many samples (> 10). We included the Cascades Mountain range (gray polygon) to serve as a landmark commonly used to differentiate where Roosevelt elk (west of cascades) and Rocky Mountain elk (east of cascades) are found. This differentiation was used as the proxy for a longitudinal gradient in models. Most samples were collected in Washington (n = 41) followed by California (n = 14), Idaho (n = 13), and Oregon (n = 6). Samples missing specific county data (n = 6) were omitted from visualization, but generally, five samples from Washington originated west of the Cascades (west) and one sample came from northern Idaho (east). Figure S2. Correlation plots and principal components analysis (PCA) biplot of available trace minerals. Left: Colored square panels show pairwise Spearman’s rank correlation coefficients (ρ) for all mineral pairs. Co-Mg, Co-Fe, Co-Mn, and Mn-Mg were the combinations with strong (≥ |0.7|) correlation values. Right: Results from PCA identifies overlapping ellipses for each U.S. state (colored ellipses) while bidirectionality in loadings suggests closely aligned vectors have similar variance structures and may be correlated (e.g., chromium and cobalt). Figure S3. Multinomial models for lesion severity in 20 TAHD positive elk. Two panels show results from multi [file 12917_2022_3547_MOESM1_ESM.docx]

# Table S1: Relative model performance given candidate datasets. We evaluated changes in relative model performance using the same model applied to different datasets with and without notable outliers removed. Note the simple model had higher area under the receiver operating characteristic curve (AUC) when outliers were removed, but sensitivity and specificity were more balanced when both outliers were removed.

| **Candidate datasets** | ***n*** | **AUC** | **Sensitivity** | **Specificity** |
| --- | --- | --- | --- | --- |
| Full dataset | 74 | 0.59 | 0.26 | 0.93 |
| Se outlier removed | 73 | 0.68 | 0.93 | 0.37 |
| Co outlier removed | 73 | 0.59 | 0.73 | 0.44 |
| Se and Co outliers both removed | 72 | 0.67 | 0.93 | 0.42 |

**
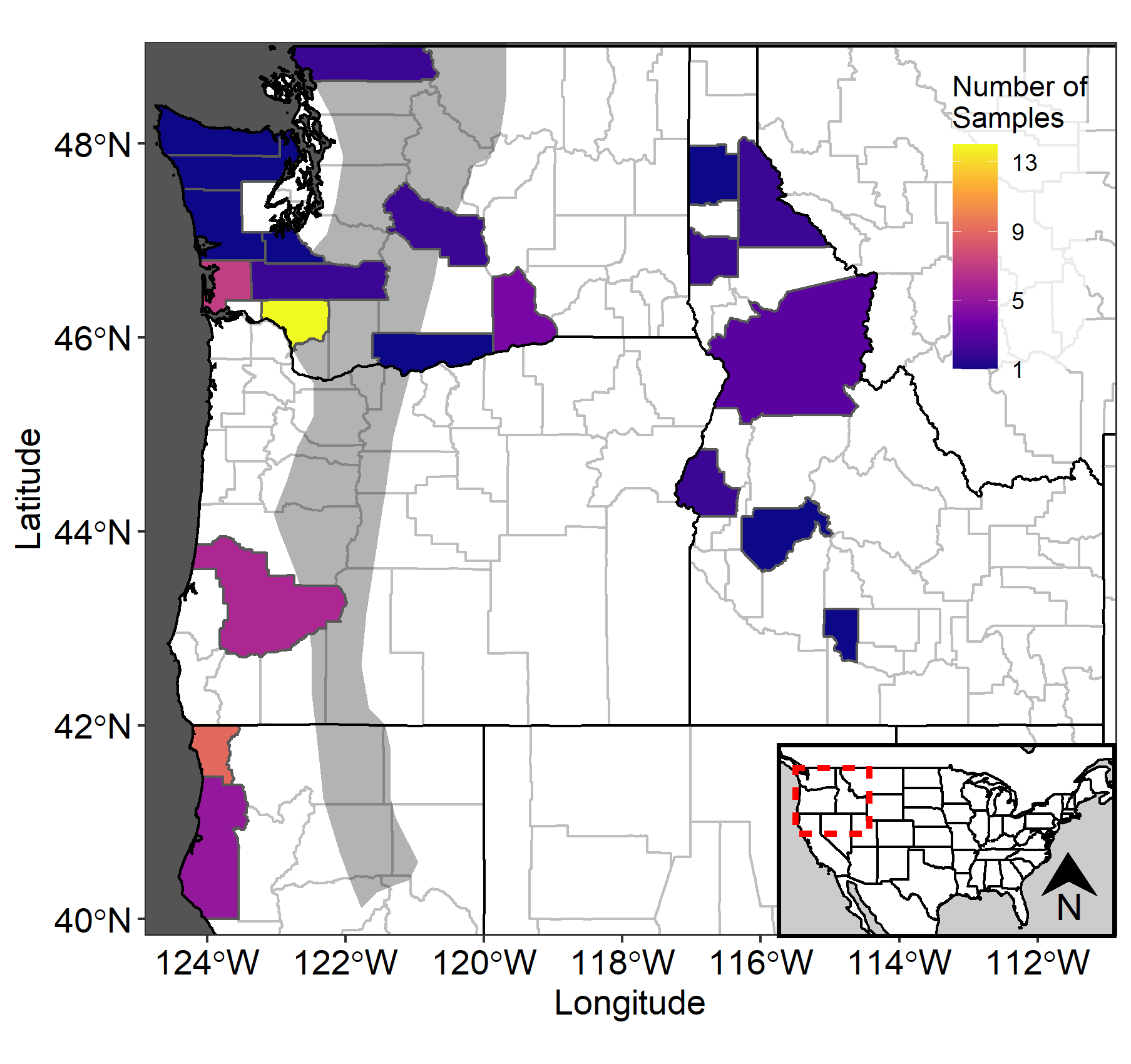
**

# **Figure S1: County-level distribution of elk hair samples for mineral analyses.** The number of samples analyzed for mineral concentrations from counties are represented by a color gradient, with dark purple colors for one to a few samples and brighter yellow colors for many samples (>10). We included the Cascades Mountain range (gray polygon) to serve as a landmark commonly used to differentiate where Roosevelt elk (west of cascades) and Rocky Mountain elk (east of cascades) are found. This differentiation was used as the proxy for a longitudinal gradient in models. Most samples were collected in Washington (n=41) followed by California (n=14), Idaho (n=13), and Oregon (n=6). Samples missing specific county data (n=6) were omitted from visualization, but generally, five samples from Washington originated west of the Cascades (west) and one sample came from northern Idaho (east).

**
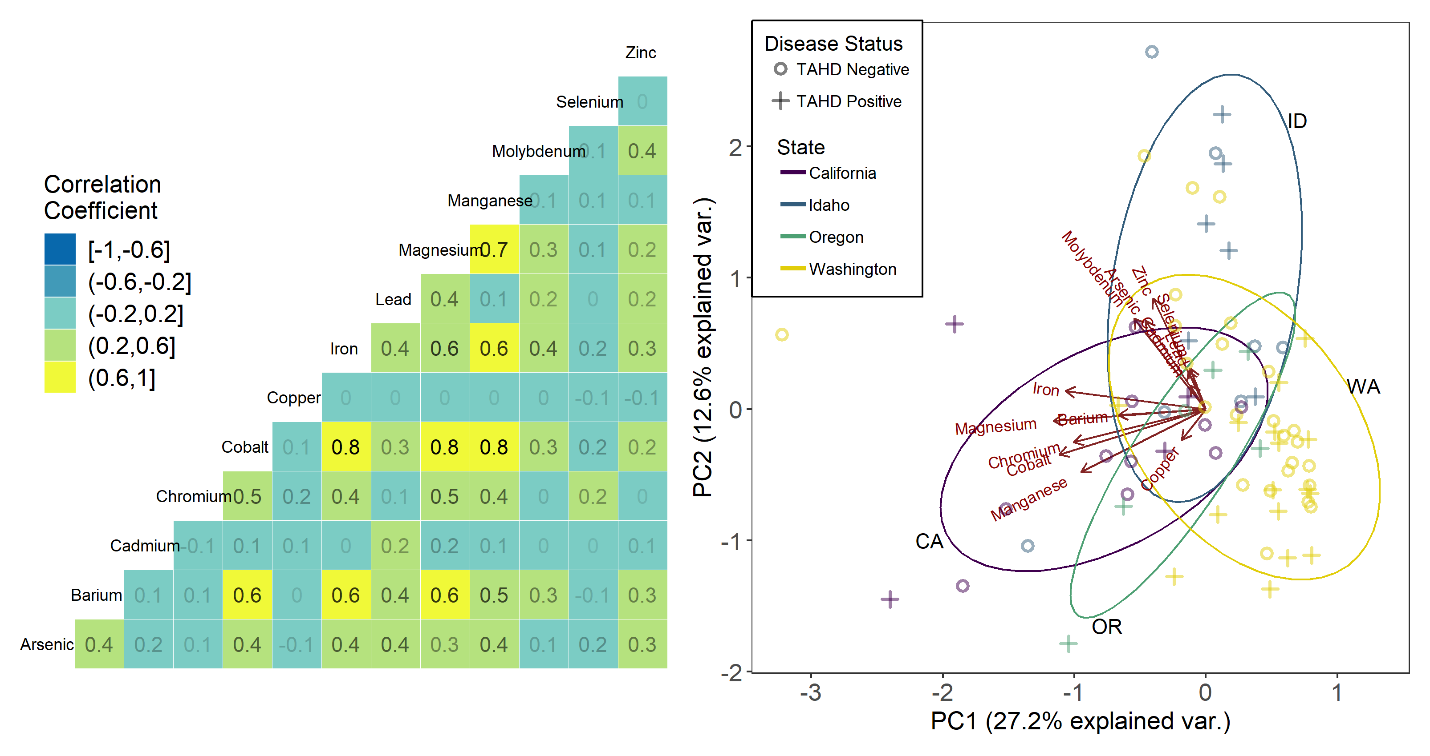
**

# Figure S2: Correlation plots and principal components analysis (PCA) biplot of available trace minerals. Left: Colored square panels show pairwise Spearman’s rank correlation coefficients (ρ) for all mineral pairs. Co-Mg, Co-Fe, Co-Mn, and Mn-Mg were the combinations with strong (≥|0.7|) correlation values. Right: Results from PCA identifies overlapping ellipses for each US state (colored ellipses) while bidirectionality in loadings suggests closely aligned vectors have similar variance structures and may be correlated (e.g., chromium and cobalt).


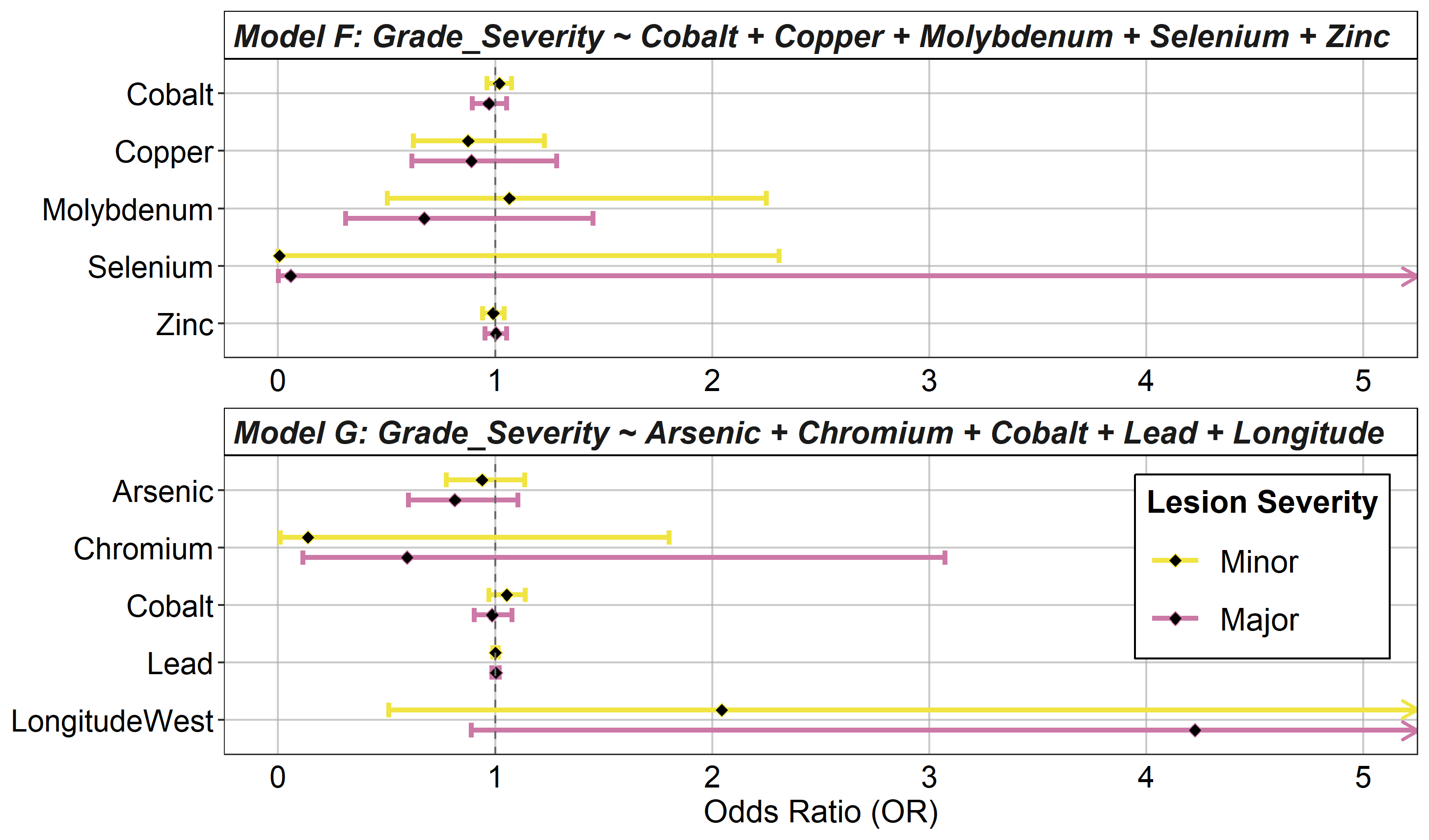


# Figure S3: Multinomial models for lesion severity in 20 TAHD positive elk. Two panels show results from multinomial models assessing severity of lesions detected: Grades I and II = Minor (yellow) and Grades III and IV = Major (pink). Odds ratios (OR; black dots) and 95% confidence intervals are graphically represented in each panel for models addressing different hypotheses (text in bold). Presumably due to the small sample size (*n*=20), we found no statistically significant evidence for odds of grade severity being influenced by minerals included under the models.


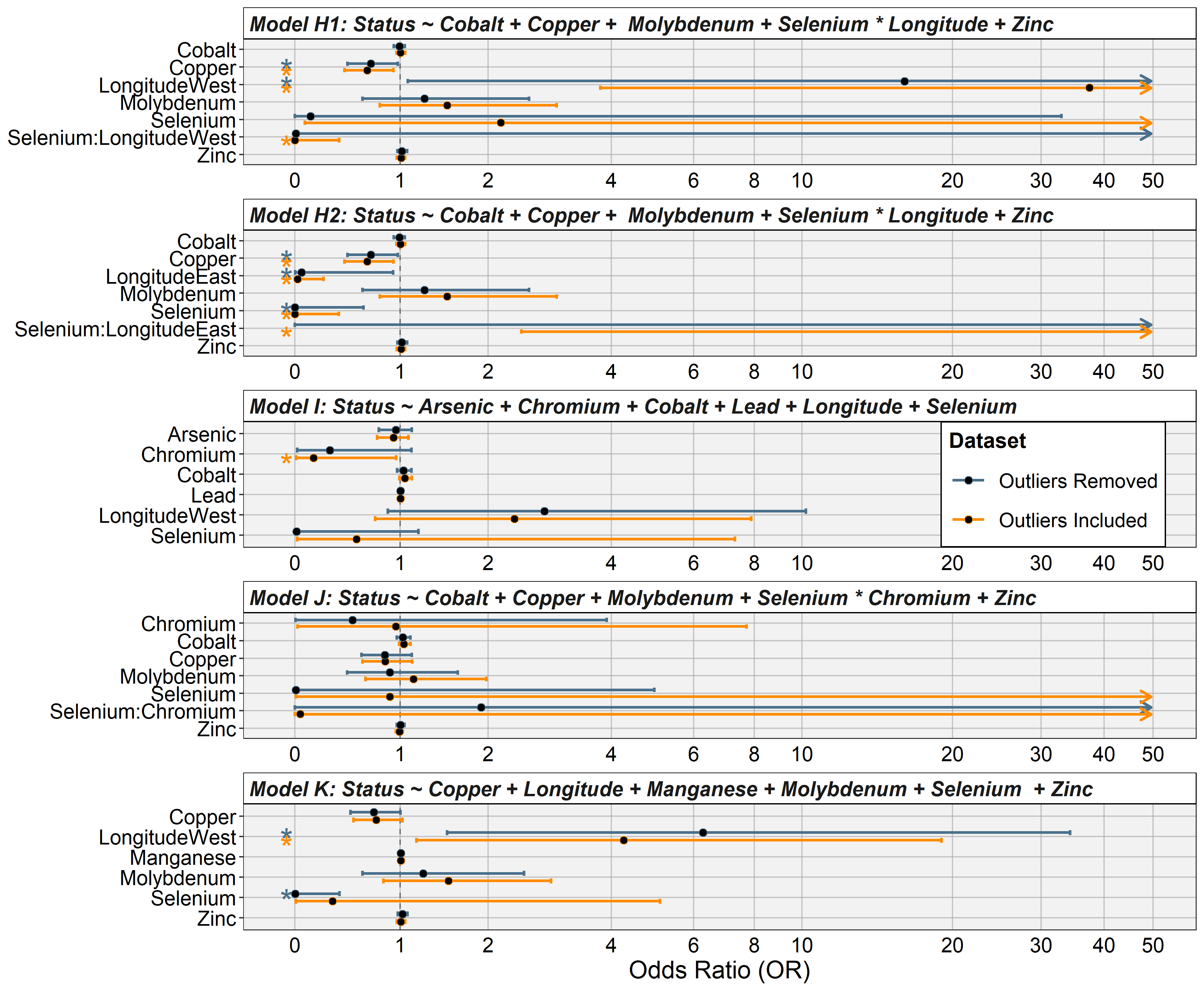


# Figure S4: Results from post hoc models. Five panels show different four different models written and visual summaries of explanatory variables’ estimates of odds ratios (black circles), 95% confidence intervals (bars), and presence of a statistically significant relationship (denoted by a star, $\boldsymbol{\star}$) color-coded to represent different datasets: outliers removed (blue) or outliers included/full dataset (orange). Please note Models H1 and H2 are identical models with different references used in the categorical Longitude variable. That is, Model H1 assesses a selenium interaction with respect to the west, while Model H2 assesses the east.
